# The Relationship of Cultivars, Structures and In Vitro Digestibility of Chinese Chestnut (*Castanea henryi*) Starches Treated with Low-Voltage Electrostatic Field Technology

**DOI:** 10.3390/molecules31213900

**Published:** 2026-10-29

**Authors:** Feng Chen, Xiantong Wu, Wencan Luo, Jing Wang, Weijing Zhuang, Wensheng Chen, Xu Lu

**Affiliations:** 1 College of Modern Agricultural Technology, Xiangsi Ling, Fujian Vocational College of Agriculture, Fuzhou 350119, China; chenfengsf@126.com (F.C.); makdwang@sina.com (J.W.); wscdsad3948@163.com (W.C.); 2 College of Food Science, Fujian Agriculture and Forestry University, Fuzhou 350002, China; 19959901736@163.com (X.W.); janey8157@sina.com (W.Z.); 3 Fujian Industrial Product Production License Review Technology Center, Fuzhou 350002, China; luowencan@163.com

**Keywords:** *Castanea henryi*, chestnut starch, LVEF, structure, gelatinization, digestibility

## Abstract

Low-voltage electrostatic field (LVEF) treatment is an emerging food preservation technology that exploits the physical effects of electrostatic fields; however, its mechanisms of action in nut crops remain poorly understood. This study investigated changes in starch structure, processing properties, and in vitro digestibility in five chestnut cultivars (Castanea henryi) from different regions of China (Chushuhong, Changmangzi, Youzhen, Hongzhenzi, and Huangzhen) following refrigeration treatment (RT) and LVEF treatment. LVEF increased the crystallinity and molecular order of chestnut starch. It also generally reduced free water content, peak viscosity (PV), breakdown viscosity (BV), and setback viscosity (SV), while increasing water-holding capacity. In addition, LVEF enhanced the resistance to shear and retrogradation, thereby improving starch processing performance. The TPA results showed that LVEF improved the textural properties of chestnut starch gels, supporting their suitability for gel-based foods. The responses varied among the cultivars. Compared with RT, LVEF reduced the particle size and free water content of Chushuhong starch by 19.56% and 18.41%, respectively. Chushuhong also exhibited the largest increase in crystallinity (31.34%), while the bound water content of its starch gel increased by 13.70%, indicating stronger responsiveness to LVEF. Overall, LVEF improved the processing performance of chestnut starch, although varietal differences in the responses were observed. LVEF-treated Chushuhong and Changmangzi starches exhibited favorable processing properties, indicating their suitability for developing processed chestnut products. Meanwhile, their starches exhibited faster enzymatic hydrolysis rates under in vitro conditions. These findings provide a basis for applying LVEF in developing chestnut starch-based products.

## 1. Introduction

Chestnuts are traditional nut crops native to China that are now widely cultivated and consumed throughout Asia, Europe, and North America [1]. Unlike many high-fat nuts, chestnuts provide energy primarily in the form of starch and are rich in protein, lipids, vitamins, and diverse bioactive compounds. Their distinctive flavor and nutritional value contribute to their widespread consumption [2]. Starch is the predominant component of chestnut dry matter, accounting for approximately 50%. In addition to being consumed fresh, chestnuts are processed into a wide range of products and used as ingredients in bread, nut-based beverages, fruit wines, and fillings, where they enhance the sensory characteristics and flavor. Chestnuts are also valued for their medicinal properties, and chestnut flour is naturally gluten-free, making it a suitable energy source for individuals with celiac disease [3]. Among chestnut species, *Castanea henryi* is one of the most widely cultivated in regions south of the Yangtze River in China [4]. According to statistics released in 2020 by the National Forestry and Grassland Administration (https://www.forestry.gov.cn (accessed on 15 September 2025)), approximately 80,000 hectares of *C. henryi* are cultivated in Fujian Province, with an annual production of approximately 75,000 tons and an annual output value of several billion yuan. Thus, *C. henryi* is a traditional nut crop of substantial economic importance in Southern China [5]. Chestnut starch is also an important ingredient in baked goods, snacks, and starch syrups. Studies have shown that starches with a greater degree of A-type crystallinity and a higher relative abundance of short amylopectin chains are often digested rapidly [6]. As the predominant nutrient in chestnuts, starch strongly influences the quality and nutritional properties of both fresh chestnuts and chestnut-derived products.

Hardening is a common physiological disorder in chestnuts during refrigerated storage. Water loss and cell wall damage contribute to this disorder and may further alter the physicochemical properties of chestnut starch [7]. Unsuitable storage conditions can impair the quality of both chestnuts and their starch. Methods traditionally used to preserve chestnuts include chemical fumigant treatments (such as carbon disulfide, phosphine, and methyl bromide), low-temperature storage, controlled atmosphere preservation, irradiation technology, and ice-water immersion [8]. Methyl bromide was once the most commonly used fumigant insecticide for post-harvest chestnuts; however, it was subsequently banned because it depletes the ozone layer and poses risks to human health [9]. Thermal treatments may require prolonged processing, whereas cold- or hot-water immersion can alter the chemical composition of chestnuts and promote mold growth [8]. Therefore, appropriate storage methods are required to preserve the quality of chestnuts and their starch.

In recent years, nonthermal physical technologies, including pulsed light, plasma treatment, and ultrahigh-pressure processing, have shown considerable potential for postharvest preservation of agricultural products [10]. Low-voltage electrostatic field (LVEF, output voltage ≤ 2500 V) is a nonthermal food preservation technology whose action is based primarily on the physical effects of electrostatic fields [11]. The applied external electric field acts on charged particles within food and can interfere with physiological and biochemical processes, thereby inhibiting microbial growth and maintaining product quality [12]. Compared with high-voltage electrostatic field (HVEF) treatment, LVEF treatment offers lower energy consumption and equipment costs, simpler operation, and greater operational safety [13], making it a promising option for food storage and preservation. Previous studies have primarily focused on the use of LVEF to preserve meat and fresh fruits and vegetables. Electric field (EF)-assisted treatment of arrowheads delayed quality deterioration by alleviating chilling injury induced by freeze–thaw cycling (FTC) [14]. Enay et al. reported that moderate electric field (MEF) treatment altered the short-range molecular order of yam starch, as indicated by increases in the Fourier transform infrared (FTIR) band ratios at 1047/1022 cm^−1^ (1.272–1.277) and 995/1022 cm^−1^ (0.724–0.734) [15]. However, research on the application of LVEF to nuts, including chestnuts, remains limited. In particular, the effects of LVEF on the postharvest preservation of different *C. henryi* cultivars and cultivar-dependent responses associated with differences in starch physicochemical properties have not been reported.

Based on these findings, we hypothesized that LVEF modulates the short-range molecular order, crystalline structure, gelatinization properties, and thermal properties of starch from different *C. henryi* cultivars, thereby altering its physicochemical characteristics and in vitro digestibility. We further hypothesized that these effects would differ significantly between cultivars. To test these hypotheses, we investigated the changes in the structure, physicochemical properties, and in vitro digestibility of starch from different *C. henryi* cultivars following LVEF treatment. Analyses were performed using nuclear magnetic resonance imaging, X-ray diffraction, Fourier-transform infrared spectroscopy, rapid viscosity analysis, differential scanning calorimetry, and texture analysis. This study extends the application of LVEF technology to nut crops and provides a scientific basis for developing food formulations with *C. henryi* starch.

## 2. Results

### 2.1. Amylose Content of Chestnut Starch

**Figure 1:**
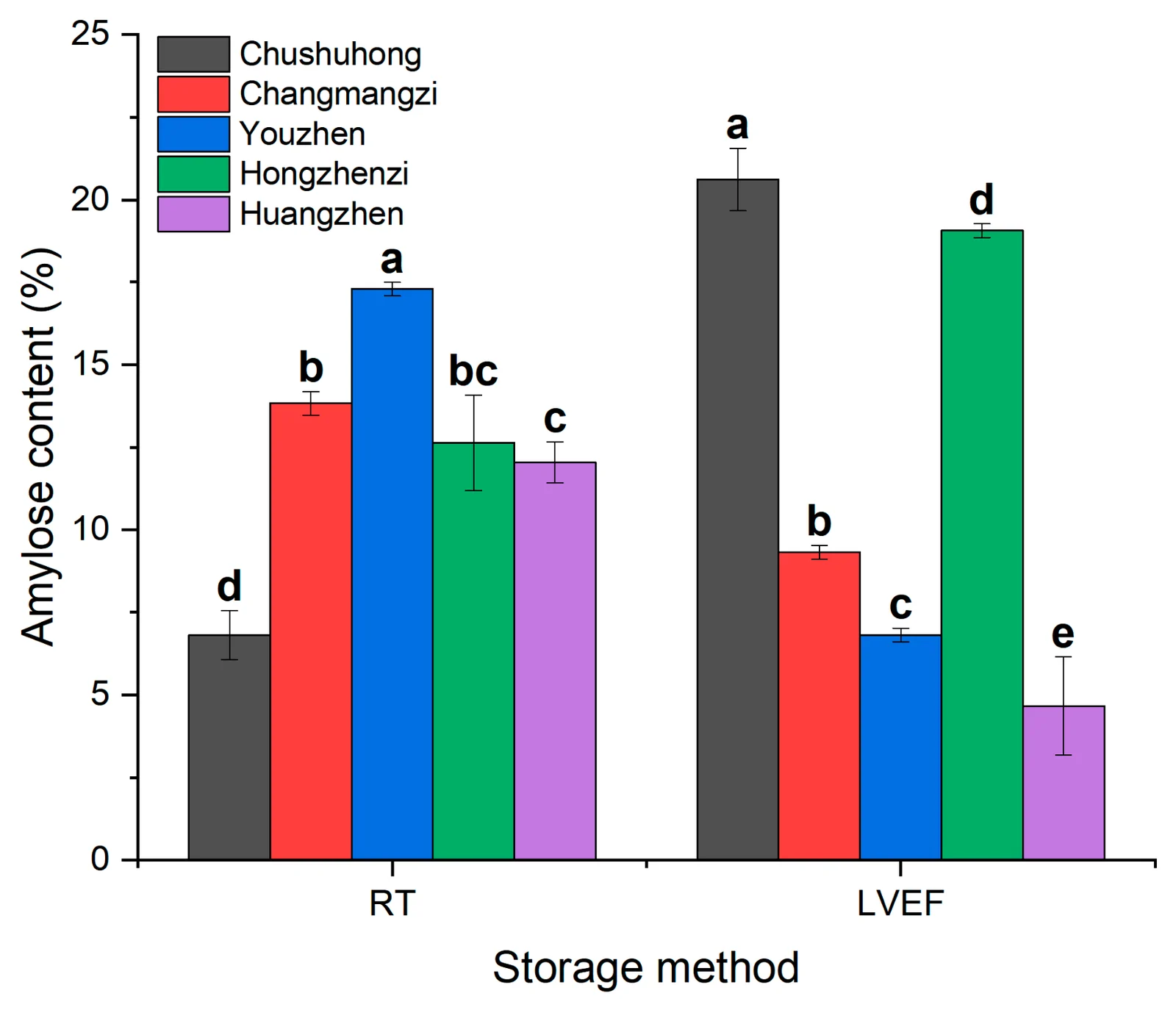
Amylose content of starches isolated from five Chinese chestnut cultivars under different storage methods. RT, refrigeration treatment; LVEF, low-voltage electrostatic field treatment. Different lowercase letters indicate significant differences in the amylose content of different chestnut starch cultivars under the same storage method (*p* < 0.05).

The amylose contents of the starches isolated from the five *C. henryi* cultivars are presented in Figure 1. Amylose content strongly influences the functional properties of starches [16]. Following LVEF treatment, the amylose contents of Changmangzi, Youzhen, and Huangzhen starches were lower than those of the corresponding RT groups; conversely, Chushuhong and Hongzhenzi contained more amylose than their respective RT counterparts after LVEF treatment. We therefore speculate that the amylose content during storage is mainly regulated by two competing processes. On the one hand, the biosynthesis of amylose depends on several classes of enzymes, including granule-bound starch synthases, starch-branching enzymes, soluble starch synthases, and starch debranching enzymes [17,18,19]. These enzymes can cleave amylopectin chains to produce amylose fractions detectable by the iodine method, thereby increasing amylose content; on the other hand, free linear amylose readily forms reorganized ordered structures and reconstructed gel networks containing stable double-helical conformations and densely packed crystalline regions during retrogradation and digestion [20]. Such components, after ordered assembly, can reduce the apparent amylose content determined by the iodine colorimetric method. For Changmangzi, Youzhen and Huangzhen, the amylose content decreased after LVEF treatment, while the RVA results showed that their setback viscosity did not increase significantly (Table 1). This suggests that this change is mainly not caused by the consumption of amylose participating in retrogradation after LVEF storage; instead, it is hypothesized that LVEF may suppress fruit respiratory metabolism, which could lower the energy supply for endogenous enzymes involved in amylose biosynthesis and consequently reduce amylose production [21]. Previous research has suggested that electrostatic fields can suppress respiration by disrupting iron ion metabolism and electron transport in the respiratory chain [22]. Liu et al. [23] similarly reported that LVEF treatment delayed the respiratory peak of crops during storage and significantly reduced respiration intensity. The resulting reduction in energy availability during LVEF storage could decrease the activity of endogenous enzymes involved in amylose synthesis, thereby lowering the amylose content. In contrast, for Chushuhong, the amylose content increased substantially after LVEF treatment, which is consistent with the pronounced decrease in SV (Table 1), indicating that retrogradation was suppressed. The amylose in Chushuhong was not extensively consumed to form iodine-inaccessible double helices, and therefore the apparent Am value was higher. This contrasting response may reflect the differences in geographical origin and fruit maturation period among the cultivars (Table S1). Within each storage treatment, the amylose content differed significantly among the cultivars (*p* < 0.05). The climate, soil type, and granule size distribution during plant development may directly influence the amylose content in chestnut cultivars [24,25].

### 2.2. Particle Size of Chestnut Starch

**Figure 2:**
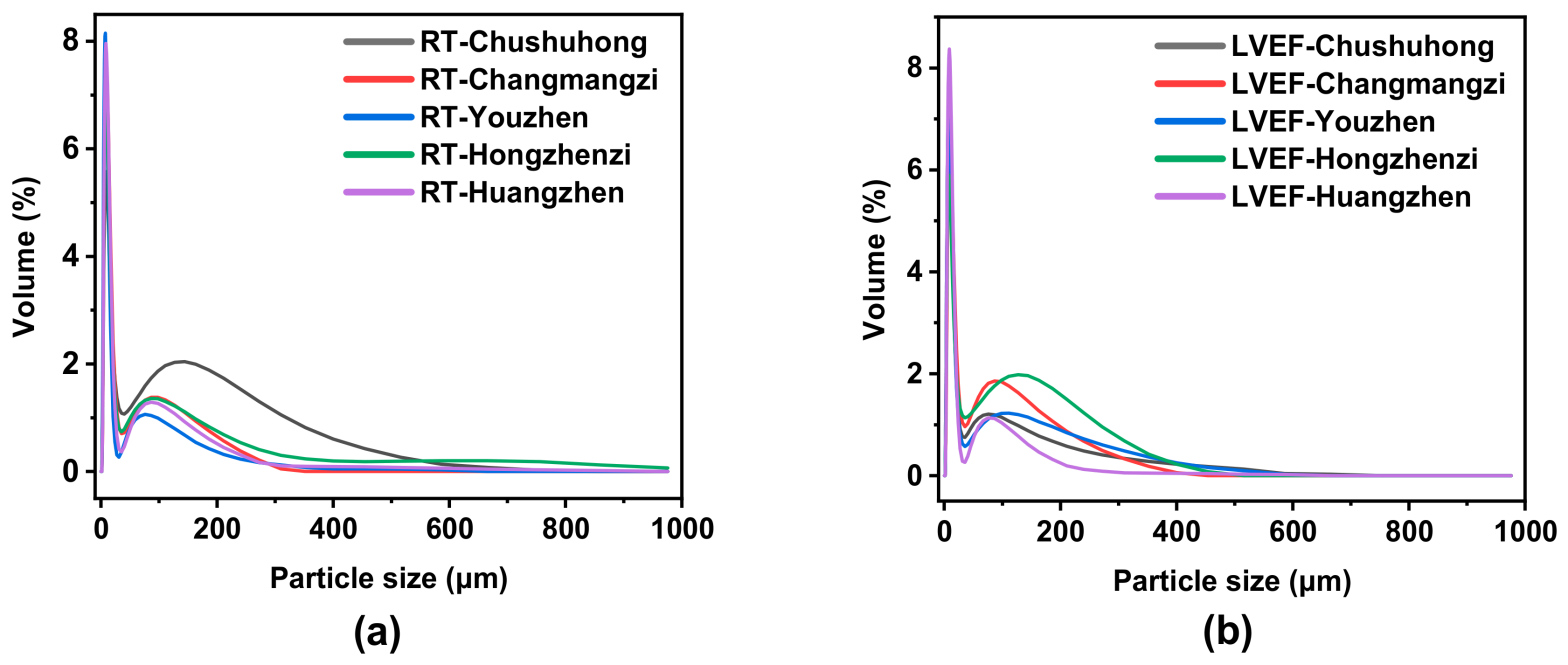
Proportion curves of the number of particles at different granularities of chestnut starch isolated from five cultivars: (**a**) RT, (**b**) LVEF. (RT: refrigerated treatment; LVEF: low-voltage electrostatic field).

Particle size is an important parameter for evaluating the quality of starch. Variations in the particle size can alter the starch structure and consequently affect its functional properties [26]. This study examined the surface-area-weighted mean particle diameter, D [2,3], and particle size distributions of starches from the five chestnut cultivars (Table 2 and Figure 2). The values ranged from 8 to 11 μm. Following LVEF treatment, the starch particle sizes of Chushuhong and Huangzhen decreased; in particular, the particle size of Chushuhong starch decreased by 19.56%, whereas Changmangzi, Youzhen, and Hongzhenzi exhibited an opposite trend. Smaller starch particles generally exhibit greater water permeability and hydrate more rapidly during thermal processing, whereas larger particles hydrate more slowly. Previous studies have demonstrated that electric-field treatment under certain conditions can distort starch-granule morphology, while the subsequent aggregation of partial granule fragments leads to larger particle size [27]. Cultivar and geographical origin also significantly influence the properties of starch [28]. Genotype-dependent differences may lead to variations in the aggregation behavior of fragmented starch granules among different chestnut cultivars. Granule fragmentation appeared to predominate in Chushuhong and Huangzhen starches, resulting in smaller apparent particle sizes. In contrast, LVEF treatment may have induced granule fragmentation followed by aggregation in Changmangzi, Youzhen, and Hongzhenzi starches, thereby increasing their particle sizes. The particle-size distributions of the chestnut starches were predominantly bimodal, with two principal peaks occurring within the ranges of 5–11 μm and 40–300 μm, respectively (Figure 2). Following LVEF treatment, the number fractions of particles within the 40–300 μm range increased significantly in Changmangzi, Youzhen, and Hongzhenzi starches, accompanied by a rightward shift in the larger-particle peak, indicating a modest increase in particle size. These distributional changes were consistent with the values presented in Table 2. Generally, a lower amylose content corresponds to a higher relative proportion of amylopectin. Additional double helical structures are formed between amylopectin side chains, and intermolecular entanglement between amylose and amylopectin is enhanced. Together, these effects contribute to an increase in the starch granule size [29,30]. Notably, both the amylose content and particle size of Hongzhenzi starch increased, deviating from this general trend. Electric field treatment may also alter the conformation of amylopectin chains within starch granules. In Hongzhenzi starch, granule swelling induced by structural changes in amylopectin may have predominated [31], masking the particle size-reducing effect associated with the increased amylose content. Previous studies have investigated the use of chestnut flour in cakes, noodles, and other flour-based products [32,33]. Chestnut starch with smaller particles can disperse more uniformly within the dough, contributing to a finer and smoother texture in baked products. Therefore, characterizing the chestnut starch particle size may inform the production and processing of chestnut flour products.

### 2.3. Water Distribution in Chestnut Starch Granules Assessed by LF-NMR

**Figure 3:**
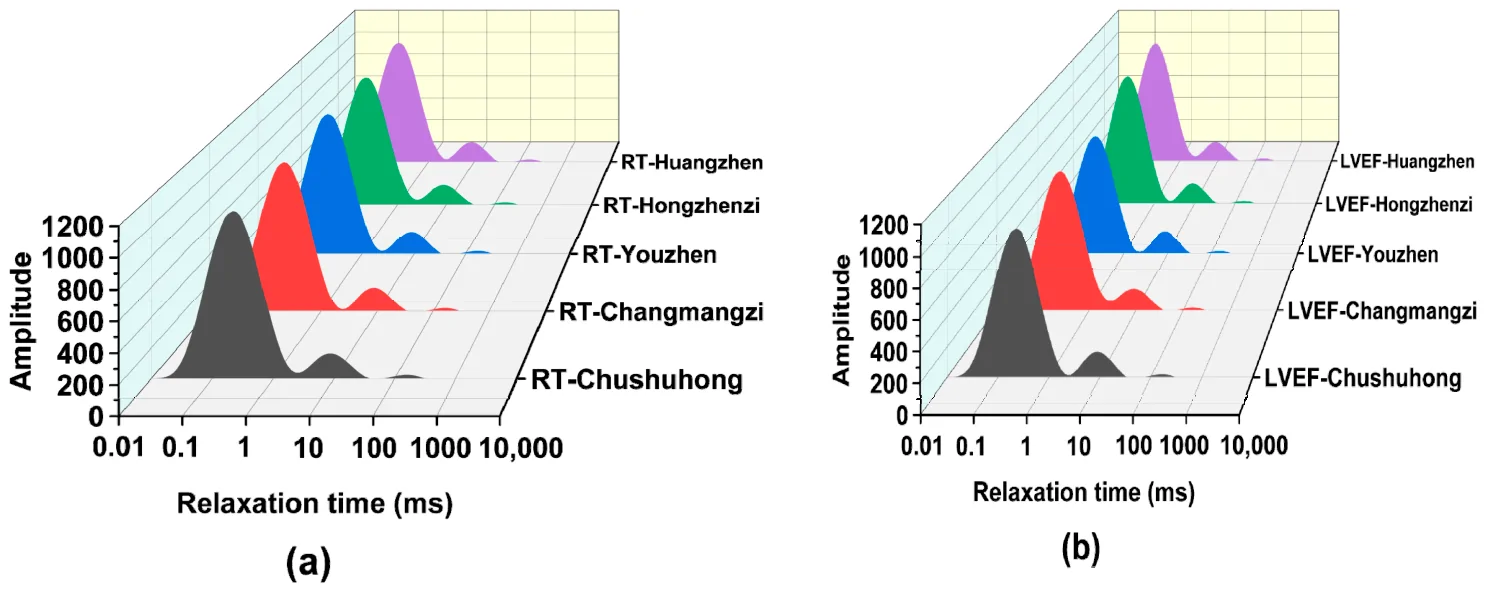
*T_2_* distribution of chestnut starch granules: (**a**) RT, (**b**) LVEF. Different colors represent different chestnut cultivars (RT: refrigerated treatment; LVEF: low-voltage electrostatic field).

Structural changes in starch can arise from water separation and displacement of starch molecules [34]. LF-NMR technology determines transverse relaxation times (*T*_2_) from molecular relaxation behavior, providing information on the mobility of water molecules within a sample. Higher *T*_2_ values indicate greater water mobility. The integrated area under each relaxation peak reflects the relative abundance of the corresponding water population. Table 2 and Figure 3 show the relative peak areas and relaxation curves corresponding to the *T*_21_, *T*_22_, and *T*_23_ components of starches from the different chestnut cultivars under the two storage conditions. Three distinct relaxation peaks were observed for all starch samples. The transverse relaxation components *T*_21_ (0.1–1 ms), *T*_22_ (1–100 ms), and *T*_23_ (100–1000 ms) were assigned to bound water, semi-bound or intermediate-state water, and free water, respectively [35]. The relaxation times were comparable among the five cultivars under both storage conditions, indicating similar water mobilities. Table 2 also shows that the relative proportions of bound, semi-bound, and free water in *C. henryi* starch are approximately 90%, 9%, and 0.7%, respectively. Differences among cultivars were only observed under LVEF storage, while no significant differences were detected among cultivars under RT storage (*p* > 0.05). Compared with RT, LVEF-treated starches generally exhibited lower free water content, accompanied by the redistribution of free water to bound or semi-bound states. This shift indicates that LVEF treatment increased the water-binding capacity of chestnut starch. This change may be attributable to the electric-field-induced polarization of the starch granules [36]. This polarized state can enhance the adsorption of water molecules by starch granules, thereby increasing the bound water fraction. Compared with RT, LVEF treatment reduced the free water content of Chushuhong starch by 18.41%, representing the largest reduction among the cultivars and helping to inhibit microbial growth. Notably, LVEF-treated Chushuhong starch had the highest amylose content, smallest granule size, and lowest free water content. These findings further support the close association between amylose content, granule size, and water distribution. The higher amylose content of Chushuhong starch was associated with smaller granules, which provided a larger relative contact area with water molecules, consequently increasing water adsorption.

### 2.4. The Long-Range Ordered Structure of Chestnut Starch Granules (XRD)

**Figure 4:**
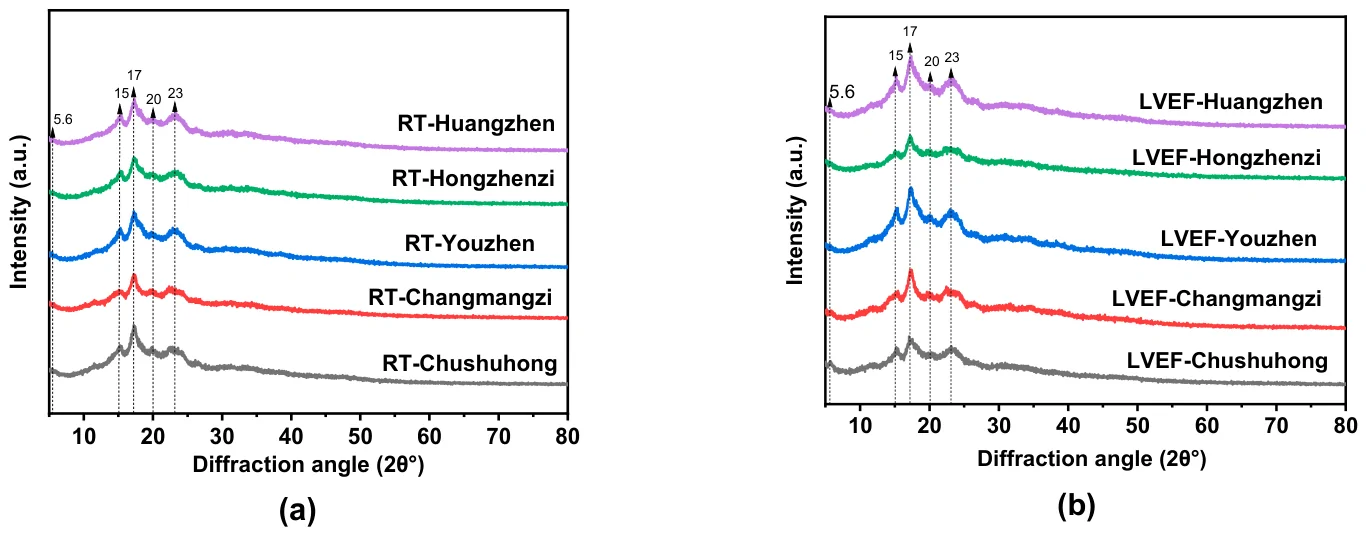
XRD patterns of chestnut starch granules isolated from five cultivars. (**a**) RT, (**b**) LVEF. (RT: refrigerated treatment; LVEF: low-voltage electrostatic field).

The XRD patterns of the five types of chestnut starch stored at RT and under LVEF storage are shown in Figure 4. Based on previous studies on the classification of starch crystal types, combined with XRD spectra, it can be determined that chestnut starch exhibits a typical C_B_-type crystalline structure, with the strongest diffraction peak near 17° 2θ, a characteristic peak around 5.6° 2θ, and several small peaks near 15° and 24°, which is consistent with previous studies on the crystalline form of chestnut starch [37,38]. As shown in Table 2, there were significant differences (*p* < 0.05) in crystallinity, sub-crystallinity, and the proportion of amorphous regions among the five types of chestnut starch stored using the same method. Overall, the crystallinities of Chushuhong and Changmangzi were relatively high. Compared to RT storage, the diffraction peaks of chestnut starch after LVEF treatment did not appear or disappear, indicating that LVEF treatment had no influence on the crystalline form of the starch. Additionally, the overall crystallinity showed an upward trend, with Chushuhong starch exhibiting the highest increase in crystallinity, increasing by 31.34%, suggesting that LVEF treatment increased the long-range order of chestnut starch relative to RT storage. It is speculated that the amylase-catalyzed hydrolysis of starch produces shorter branched starch that can bind water molecules and suppress the evaporation of water molecules that would decompose the starch chains, thereby inhibiting molecular disorder [39]. This is consistent with the results of a previous study on the moisture distribution of chestnut starch granules.

### 2.5. Short-Range Order of Chestnut Starch Granules Assessed by FTIR

**Figure 5:**
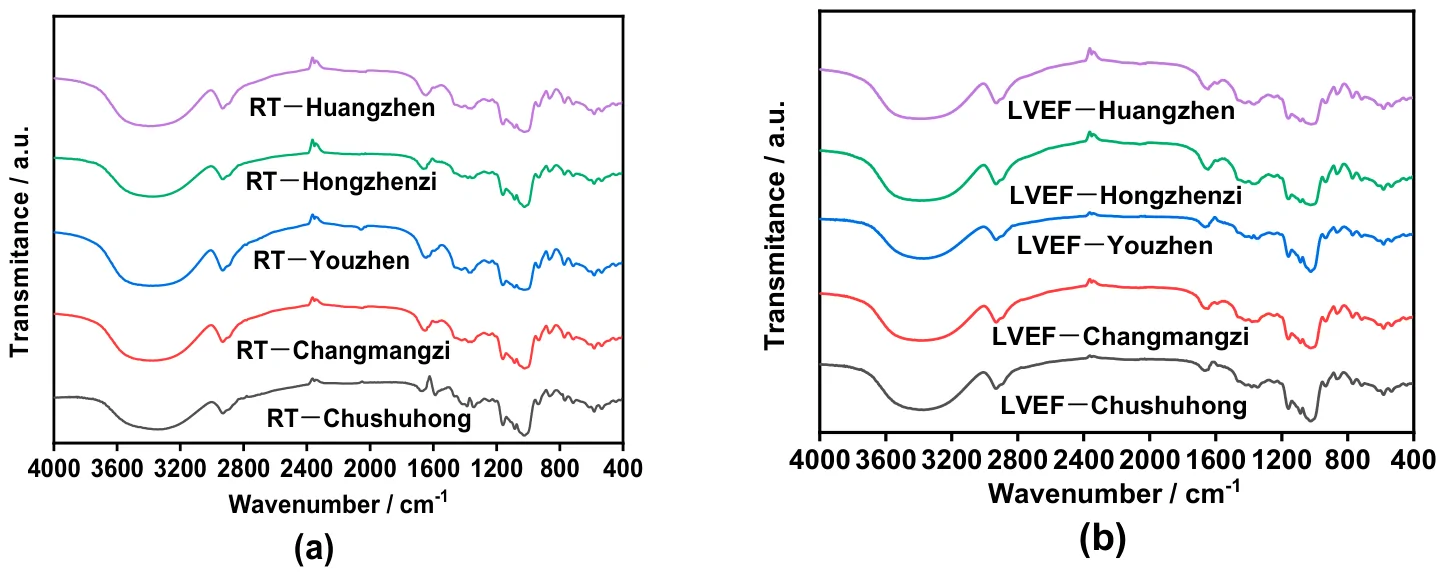
FTIR spectra of chestnut starch granules isolated from five varieties. (**a**) RT, (**b**) LVEF. (RT: refrigerated treatment; LVEF: low-voltage electrostatic field).

FTIR spectroscopy was used to characterize the short-range molecular order of starch isolated from the five chestnut cultivars. Compared to RT, LVEF treatment produced no new absorption bands in any starch sample (Figure 5). This finding indicates that LVEF did not alter the chemical functional groups present in starch and that the observed changes were physical rather than chemical. All samples exhibited a broad, relatively intense absorption band at 3200–2800 cm^−1^, which was attributed to the O-H stretching vibrations of glucose units within the starch molecules [40]. The R_1047/1022_ and R_995/1022_ band intensity ratios are indicators of short-range starch order and reflect the degree of double helical organization. Higher ratio values correspond to a greater molecular order within the starch granules [41]. As shown in Table 2, both ratios differed significantly among the chestnut cultivars (*p* < 0.05). Following RT storage, Changmangzi starch exhibited the highest R_1047/1022_ and R_995/1022_ values, indicating greater short-range double-helical order than the other cultivars. This greater molecular order suggests stronger intermolecular and intramolecular hydrogen bonding within the Changmangzi starch matrix, which is consistent with the XRD results (Table 2). Following LVEF treatment, chestnut starches showed an overall increase in both R_1047/1022_ and R_995/1022_, indicating an enhanced short-range molecular order. The electric field may initially act on polar groups, such as hydroxyl groups, disrupting the electrostatic balance of intermolecular and intramolecular hydrogen bonds and weakening the hydrogen bond network. The dispersed starch chains may subsequently rearrange under the combined electric field and low-temperature conditions to form stronger hydrogen bonds and a denser molecular structure, ultimately increasing the short-range molecular order [36,42]. Short-range molecular organization differed markedly among the chestnut cultivars and may contribute to the differences in their physicochemical properties. These cultivar-dependent differences provide opportunities to broaden the applications of chestnut starch and warrant further investigation into its functional properties.

### 2.6. Pasting Properties of Chestnut Starch Assessed by RVA

**Figure 6:**
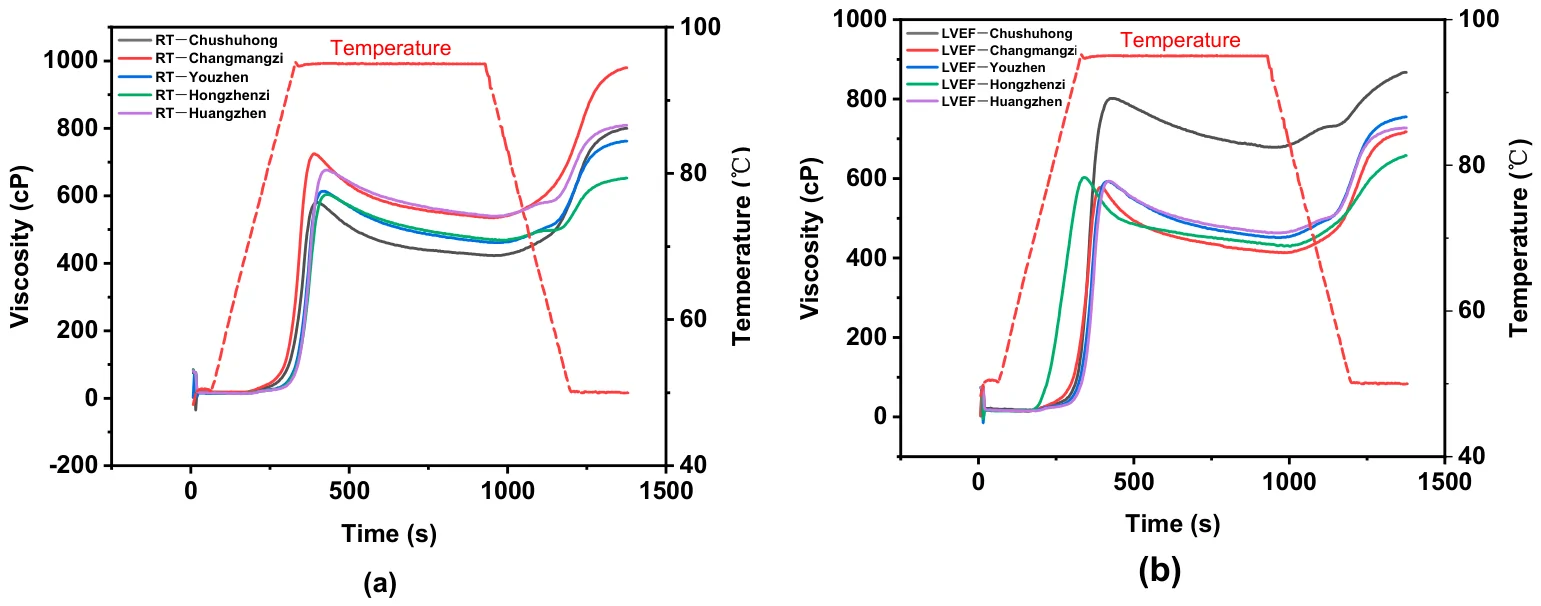
RVA curve of chestnut starch granules isolated from five varieties. (**a**) RT, (**b**) LVEF. (RT: refrigerated treatment; LVEF: low-voltage electrostatic field).

Starch gelatinization involves the absorption of water by starch granules, leading to their swelling and molecular diffusion, which increases viscosity. Retrogradation occurs when gelatinized starch molecules reassociate, and the starch paste forms a gel. Rapid viscosity analysis (RVA) characterizes the pasting behavior of starch in water by measuring the changes in viscosity during gelatinization and subsequent molecular reassociation. The resulting pasting parameters are important for the production of starch-based foods because they reflect the swelling behavior of starch granules. The pasting profiles of starches from the different chestnut cultivars following RT or LVEF storage are presented in Table 1 and Figure 6. Several pasting parameters differed significantly among the cultivars (*p* < 0.05). Pasting temperature (PT) is the temperature at which the viscosity of a starch system begins to increase. In the RVA measurement curve, PT is generally identified as the inflection point at which the viscosity begins to increase markedly [43]. Following LVEF treatment, PT increased slightly or remained unchanged in most cultivars but decreased in Chushuhong and Hongzhenzi starches. For Chushuhong starch, this decrease may be related to the reduction in the particle size. Peak viscosity (PV) represents the maximum viscosity attained by gelatinized starch during heating in water, and reflects the water-holding capacity of the starch sample. Following LVEF treatment, PV decreased in all cultivars except for Chushuhong. The PV of Chushuhong starch increased and was markedly higher than that of the other four cultivars. This increase may be attributable to the reduction in particle size following LVEF treatment (Section 2.2). Compared to larger starch particles, smaller particles allow water to diffuse and penetrate more readily. In general, a lower PT and higher PV are associated with favorable food quality; accordingly, LVEF-treated Chushuhong starch exhibited favorable eating quality potential [44]. Breakdown viscosity (BV) is used to evaluate the thermal stability and shear resistance of starch; a higher BV indicates lower resistance to shear [39]. Relative to RT, LVEF treatment reduced BV in all cultivars except Hongzhenzi, indicating an increase in shear resistance. The PT of the RT-treated Changmangzi starch was 90.7 ± 0.19 °C, indicating a relatively high swelling capacity. This characteristic corresponded to a higher PV of 727 ± 3.14 cP and BV of 187 ± 0.1 cP, indicating low cooking stability [45]. Setback viscosity (SV) reflects short-term retrogradation and the capacity of amylose molecules to reassociate and rearrange within gelatinized starch [46]. A lower SV indicates greater resistance to short-term retrogradation [47]. Products formulated primarily with low-SV starch therefore harden more slowly during storage and are suitable for producing soft and sticky starch-based foods. Overall, relative to RT, LVEF treatment reduced PV and SV in most cultivars and increased the shear resistance of the starch pastes. Lower SV values indicate enhanced resistance to retrogradation.

### 2.7. Thermal Properties of Chestnut Starch Granules Assessed by DSC

**Figure 7:**
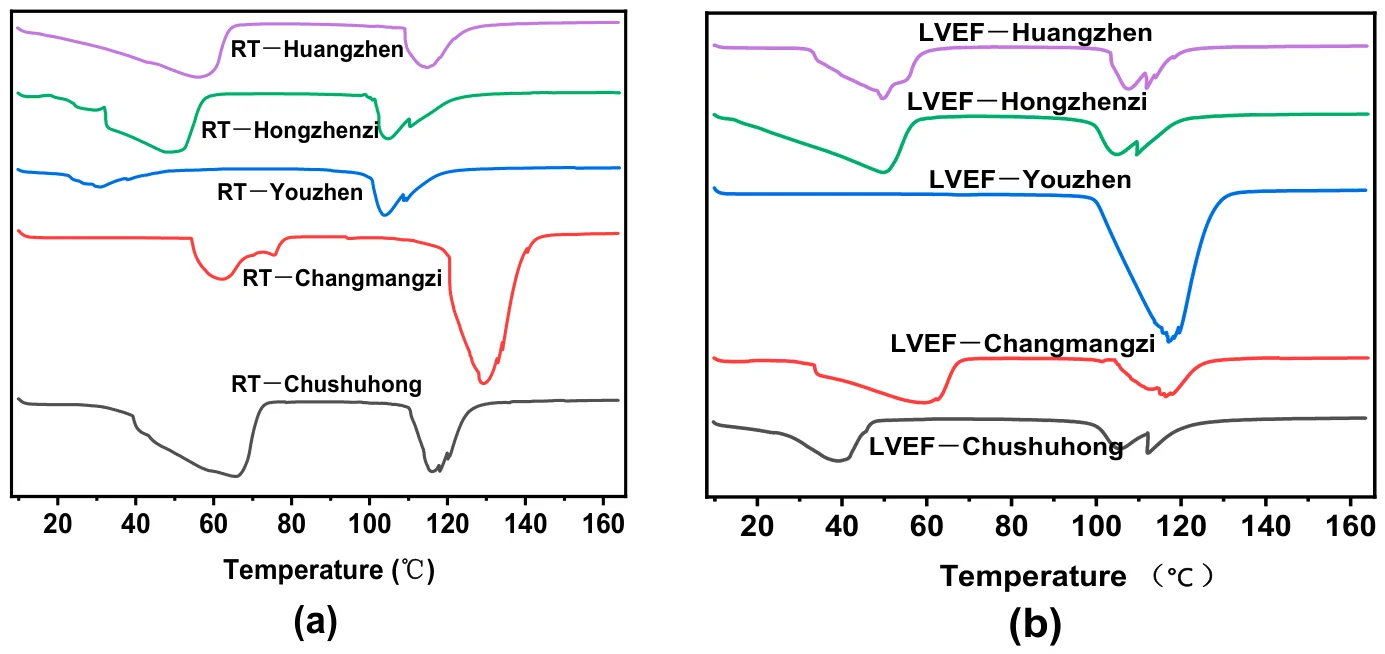
DSC curves of chestnut starch granules isolated from five varieties. (**a**) RT, (**b**) LVEF. (RT: refrigerated treatment; LVEF: low-voltage electrostatic field).

The differential scanning calorimetry (DSC) thermograms used to characterize the gelatinization behavior of chestnut starch are presented in Figure 7. Under excess water conditions (30 mg of starch mixed with 100 μL of distilled water), the first-heating DSC thermograms exhibited two endothermic peaks. The first, lower-temperature peak corresponded to starch gelatinization, during which heat was absorbed primarily to melt the less stable native crystallites as the granules absorbed water and swelled. The second, higher-temperature peak was attributed to the melting of more thermally stable ordered crystallites [38,48]. As shown in Table 3, the peak temperature (*T*_p_) and enthalpy change (*ΔH*) differed significantly among the chestnut cultivars (*p* < 0.05). Notably, the first-peak *T_p_* values of RT-Youzhen exhibited a relatively low peak temperature (31.1 ± 0.18 °C). We speculate that this abnormally low-temperature endothermic phenomenon observed in RT-Youzhen may stem from the reaction of cultivar-specific endogenous starch hydrolases on the starch structure during storage. Partial enzymatic hydrolysis may alter the integrity of starch molecular chains and ordered structures, making them more susceptible to disintegration during hydration and heating. This hypothesis is consistent with the relatively low structural order of RT-Youzhen starch. The unique starch structure of the Youzhen cultivar and its susceptibility to endogenous enzyme activity may account for its abnormal thermal behavior. Partial hydrolysis by endogenous enzymes can promote the formation of amylose, further affecting its binding with endogenous lipids (the enthalpy value in the high-temperature region increased from 5.1 J/g to 29.5 J/g, while the apparent amylose content decreased due to complex formation with lipids) and increasing the proportion of V-type crystals, indicating a substantially enhanced contribution of this thermal event. Among the RT samples, Chushuhong and Changmangzi starches had the highest *T_p_* values, consistent with their higher proportions of crystalline regions. Following LVEF treatment, the *T_p_* values of both the gelatinization and melting peaks of retrograded crystallites decreased in Chushuhong, Changmangzi, and Huangzhen starches. These decreases suggest a lower degree of starch retrogradation and greater resistance to retrogradation compared to RT storage. This response was consistent with the changes in setback viscosity described in Section 2.6, indicating that LVEF treatment improved retrogradation resistance in these cultivars. The *ΔH* value reflects changes in the ordered structures of amylopectin side chains, including crystalline and double-helical structures [49], and is generally negatively correlated with amylose content [50]. No distinct first endothermic peak and quantifiable transition enthalpy were observed in LVEF-Youzhen. However, the absence of a well-defined peak does not mean the complete disappearance of native starch crystallites. The Youzhen sample still retained crystalline diffraction patterns, and its relative crystallinity increased from 24.76% under the control (RT) condition to 25.77% after LVEF treatment. Therefore, the weak endothermic response in the low-temperature region cannot be simply attributed to complete hydrolysis of crystalline structures. XRD and DSC reflect starch structures from different perspectives: the former characterizes structural order, while the latter measures thermal transition processes under specific water content and heating conditions. The endothermic process in the high-temperature region (enthalpy of 29.5 J/g) should be considered separately. The dissociation of amylose–lipid complexes may be one of the causes of this endothermic process, owing to their more thermally stable structures. In addition, the decrease in apparent amylose content in the LVEF-Youzhen sample supports our previously proposed explanation that extensive hydrolysis increases the availability of amylose and thereby generates more complexes. Following LVEF treatment, the first peak *ΔH* decreased in Chushuhong and Huangzhen starches but increased in Changmangzi and Hongzhenzi starches, demonstrating cultivar-dependent thermal responses to LVEF.

### 2.8. Water Distribution in Chestnut Starch Gels Assessed by LF-NMR

**Figure 8:**
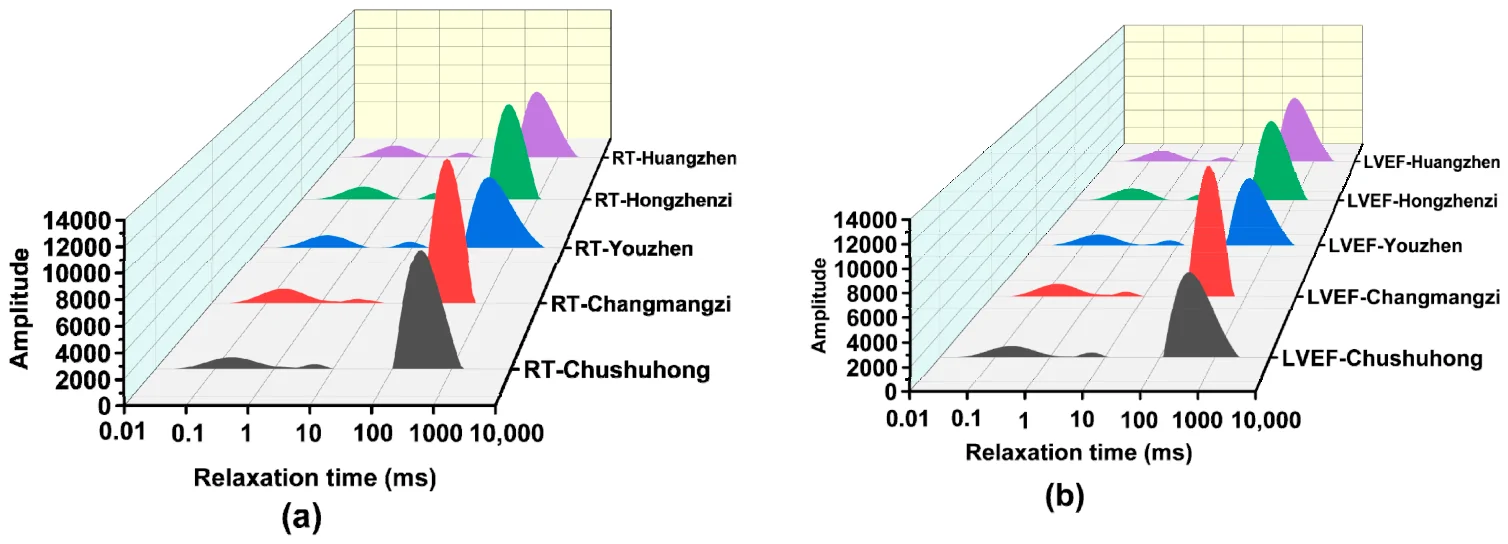
*T*_2_ distribution of chestnut starch gels: (**a**) RT and (**b**) LVEF. Different colors represent different chestnut cultivars (RT: refrigerated treatment; LVEF: low-voltage electrostatic field).

Water distribution directly influences the texture of starch gels, including their hardness, springiness, chewiness, mouthfeel (softness and juiciness), and water retention capacity. Characterizing water distribution can therefore inform improvements in product performance, process optimization, and the development of additional applications. In this study, the water distribution of gels prepared from different chestnut starches following RT or LVEF treatment was examined. The relative peak areas and relaxation curves corresponding to the *T*_21_, *T*_22_, and *T*_23_ components are presented in Table 4 and Figure 8. Bound, semi-bound, and free water accounted for approximately 12–17%, 1–4%, and 80–86% of the total water in the chestnut starch gels, respectively. As shown in Table 4, Chushuhong and Changmangzi starch gels contained higher proportions of free water and lower proportions of bound and semi-bound water than the other cultivars. The relaxation profiles in Figure 8 show markedly longer *T*_2_ relaxation times for Youzhen and Huangzhen and shorter relaxation times for Changmangzi. Longer *T*_2_ values reflect weaker interactions between water molecules and the gel network, and consequently, greater water mobility. Conversely, shorter *T*_2_ values indicate that water molecules are more strongly constrained by the gel network and have lower mobility [51]. Accordingly, Youzhen and Huangzhen starch gels exhibited relatively weak water retention, whereas Changmangzi starch gel exhibited comparatively strong water retention. Although Changmangzi starch gel contained a relatively high proportion of free water, its shorter *T*_2_ relaxation time indicated that this water was less mobile and more strongly retained within the gel network. This combination may contribute to its favorable processing. Following LVEF treatment, the *T*_23_ peak areas of Chushuhong and Hongzhenzi starch gels decreased, accompanied by a reduction in free water content. This response may be attributable to LVEF-induced polarization, which can cause the localized expansion or distortion of the helical structures of starch molecules and disrupt intramolecular hydrogen bonds. These structural changes may hinder the hydrogen-bond-mediated aggregation of starch molecules into insoluble structures, thereby reducing water expulsion from the gel network [34]. Among the cultivars, Chushuhong starch gel exhibited a 13.70% increase in bound water content and a 2.56% reduction in free water content following LVEF treatment, indicating a strong responsiveness to LVEF.

### 2.9. Texture Properties of Chestnut Starch Gels Assessed by TPA

Texture profile analysis (TPA) quantitatively characterizes the mechanical properties of food. The textural properties of starch pastes and gels are closely associated with the quality and storage stability of starch-based foods and contribute to the mechanical sensations experienced during mastication. The textural properties of the chestnut starch gels are summarized in Table 4. The gel textural properties differed significantly among the chestnut cultivars (*p* < 0.05). This result is consistent with the findings of Kong et al., who reported significant cultivar-dependent differences in the hardness, cohesiveness, and adhesiveness of starch gels [43]. These findings indicate that the cultivar strongly influences the starch-gel texture. Among the measured properties, hardness is an important determinant of consumer preferences. Previous research has shown that starch can increase the hardness of certain foods, including gummy candies [52]. Therefore, starch may be used in place of certain texturizing additives to achieve the desired product texture. The highest hardness was observed in the LVEF-treated Chushuhong starch gel (194.33 ± 15.34). High starch gel hardness can be attributed to the elevated amylose content and long amylopectin chains [53]. During retrogradation, linear amylose molecules readily reassociate, promote crystallization, and form a dense ordered network, thereby increasing gel hardness and strength [54]. In contrast, the highly branched structure of amylopectin impedes molecular reorganization, resulting in a less ordered arrangement of starch chains following gelatinization [55,56]. In addition, the smaller molecular size of amylose facilitates leaching and molecular rearrangement during gelatinization, promoting the formation of firmer gel structures [57]. Following LVEF treatment, all measured textural parameters, except springiness, increased in Chushuhong, Changmangzi, and Huangzhen starch gels, whereas the corresponding values decreased in Youzhen and Hongzhenzi starch gels. Overall, Changmangzi exhibited relatively high hardness, springiness, cohesiveness, chewiness, and resilience values. Cohesiveness reflects the resistance of the starch gel to mechanical deformation and its ability to retain structural integrity during repeated compression [58]. Higher cohesiveness indicates a more structurally intact gel, whereas lower cohesiveness indicates a more fragile structure [59]. However, Santos et al. reported that sufficiently low chewiness can reduce the energy required for mastication [60]. Therefore, cultivar selection can be tailored to the specific chewiness requirements of the intended chestnut starch product.

### 2.10. In Vitro Digestibility of Chestnut Starch

Studying the in vitro digestibility of starch can quickly and efficiently predict the digestion and absorption characteristics of starch in the human body, providing key evidence for food development and nutritional value assessment. There were significant differences (*p* < 0.05) in the RS, SDS, and RDS contents among the different chestnut starch cultivars. This may be attributed to the fact that starch digestion is influenced by various factors, such as the shape and size of starch granules, their crystallinity, amylose content, and the distribution of amylopectin branch lengths [61]. The interactions between these factors complicate the mechanism of starch digestion through enzymatic reactions [50]. Table 5 shows that the RS content of Huangzhen and Hongzhenzi starch stored at RT was relatively high, indicating that they have strong resistance to digestion. This may be because the starch granules of these two chestnuts have a higher degree of order (FTIR), while starch with a relatively irregular and unordered structure is more easily hydrolyzed; conversely [62], it is less easily hydrolyzed and digested. Elevated RS levels can retard starch hydrolysis in simulated in vitro systems. According to the in vitro digestion results, RS content decreased for most chestnut starches after LVEF treatment, suggesting an increased extent of in vitro enzymatic hydrolysis. Previous XRD and FTIR results indicated that both long- and short-range molecular order of *C. henryi* starch granules were generally increased after LVEF treatment. Nevertheless, the decreased RS content indicates that the improvement in molecular order structure of starch upon LVEF treatment does not equal enhanced enzymatic-resistance capacity. This observation may be ascribed to the fact that the electric field can disrupt starch dense structural lamellae and introduce structural defects, which improves the accessibility of starch to amylase. Consistent with this finding, Liang et al. [63] also reported that appropriate electric-field treatment could expose more enzyme-accessible sites on starch molecules, thus promoting enzymatic reaction. Similar observations have also been reported for other non-thermal physical technologies (e.g., ultrasonication): starch crystallinity and short-range order can be enhanced, whereas cavitation induces granule surface damage and micropore formation, thereby increasing amylase accessibility. It is hypothesized that electric fields exert bidirectional effects on starch crystallites: the increase in crystallinity and enzyme hydrolysis acceleration caused by granule-surface damage represent two independent competing effects. The enhanced amylase accessibility overrides the anti-digestive effect originating from crystallite ordering, ultimately leading to a reduction in RS content [64].

## 3. Discussion

Compared to RT storage, LVEF treatment was associated with lower amylose content in several chestnut cultivars. This response may arise because LVEF inhibits cellular respiration, thereby restricting energy availability and indirectly affecting the activity of endogenous enzymes involved in amylogenic synthesis [22,23]. However, Chushuhong and Hongzhenzi exhibited opposite responses. This divergence suggests that amylose content during storage is also influenced by geographical origin and the maturation period. Chushuhong originated primarily from Southern Zhejiang, whereas the other cultivars originated from Fujian Province. Chushuhong and Hongzhenzi typically mature in early to mid-September, whereas the remaining three cultivars mature later, in late September or October [24]. These inherent cultivar-related factors may partly account for the differential amylose responses to LVEF. Although LVEF may inhibit cellular respiration, it can also preserve plant cell wall integrity, thereby limiting amylose loss [25]. This protective effect may have predominated in Chushuhong and Hongzhenzi samples. LVEF treatment increased the starch particle size and the proportion of less-mobile water, comprising bound and semi-bound water, in most cultivars. These changes in water distribution may reflect the electric field-induced polarization of starch granules [14]. This polarized state can enhance the adsorption of water molecules by starch, thereby increasing the bound water fraction. The increase in less-mobile water may help maintain starch granule structural stability. Consistent with these changes, the crystallinity determined by XRD, short-range molecular order indicated by R_1047/1022_, and double-helical order indicated by R_995/1022_ generally increased following LVEF treatment. LVEF may also promote the enzymatic hydrolysis of starch, generating shorter branched chains with an enhanced water retention capacity. By maintaining water content and limiting water evaporation and starch chain disruption, this process may suppress molecular disorder [39]. This interpretation is consistent with the LF-NMR results, which showed that LVEF increased the proportion of bound and semi-bound water within the starch granules. The increase in the short-range molecular order may also result from the interaction of the electric field with polar groups, such as hydroxyl groups, within the starch molecules. This interaction may initially disrupt the electrostatic balance of intermolecular and intramolecular hydrogen bonds, ultimately weakening the overall hydrogen bond network [36]. Subsequently, the dispersed starch chains may rearrange under the combined electric field and low-temperature environment to form stronger hydrogen bonds and a denser molecular structure, which may increase the short-range order. Starch granules with greater crystallinity and short-range molecular order generally exhibit lower swelling capacities. Amorphous regions are the principal sites of amylose leaching, and starch water solubility tends to increase with the proportion of amorphous regions [65,66,67]. Following LVEF treatment, the swelling of chestnut starch granules was partially inhibited. Simultaneously, LVEF disrupted portions of the amorphous regions and promoted the leaching of some amylose molecules, thereby affecting starch water solubility. The initial weakening of hydrogen bonds, followed by a more ordered rearrangement of starch molecules, appeared to restrict starch swelling, as reflected by the lower PV values observed in most cultivars. These structural changes also increased the shear resistance of the starch pastes, as indicated by lower BV values, and enhanced their resistance to retrogradation, as indicated by lower SV values. The response of chestnut starch to LVEF treatment was cultivar-dependent. LVEF-treated Chushuhong retained a higher amylose content, indicating reduced amylose leaching from starch granules. It also exhibited a smaller granule size, greatest water-binding capacity, highest crystallinity, and most favorable pasting properties. The latter was characterized by the highest PV and lowest BV, indicating strong shear resistance and thermal stability [39], as well as the lowest SV and *ΔH*, indicating the greatest resistance to retrogradation [47]. LVEF-treated Chushuhong and Changmangzi also exhibited favorable gel texture properties. These two cultivars, therefore, showed favorable processing suitability but had higher RDS proportions and more rapid enzymatic hydrolysis rates under in vitro conditions. Under RT conditions, Huangzhen and Hongzhenzi starches exhibited relatively high RS contents and stronger in vitro anti-enzymatic hydrolysis capacity, which may be associated with their greater molecular order. These findings indicate comparatively strong resistance to enzymatic hydrolysis under simulated digestion conditions; however, they cannot be used directly to determine the in vivo glycemic index of these cultivars. These findings provide a basis for the subsequent development of chestnut-based products with low glycemic indices. This study had some limitations. Although this study has minimized potential environmental differences between the two storage devices other than the electrostatic field, such differences cannot be completely eliminated. For further verification of the present findings, future research may adopt low-voltage electrostatic field equipment that allows the electrostatic field to be switched off within the same chamber while keeping all other environmental parameters constant.

## 4. Materials and Methods

### 4.1. Materials and Chemicals

Five cultivars of chestnut (Genus Castanea, *Castanea henryi*) were used: Chushuhong, Changmangzi, Youzhen, Hongzhenzi, and Huangzhen. Chushuhong originated from the Southern region of Zhejiang Province, whereas Changmangzi, Youzhen, Hongzhenzi and Huangzhen originated from Jian’ou City, Fujian Province. All samples were collected in the same year (Table S1). Potassium bromide (KBr) was purchased from Shanghai Macklin Biochemical Co., Ltd. (Shanghai, China). Glucoamylase (100,000 U) and α-amylase (9400 U/g) were purchased from Shanghai Yuanye Biotechnology Co., Ltd. (Shanghai, China). Unless otherwise specified, all other chemicals and reagents were of analytical grade and obtained from Sinopharm Chemical Reagent Co., Ltd. (Shanghai, China).

### 4.2. Chestnut Storage and Starch Extraction

All chestnuts were sorted, and damaged fruits were discarded. Samples of each cultivar were divided equally into two groups. One group was subjected to refrigeration treatment (RT, 4 °C), whereas the other was stored under a low-voltage electrostatic field (LVEF, 4 °C). The preservation unit used for LVEF treatment (DENBA JAPAN Co., Ltd., Tokyo, Japan) combined a low-voltage electrostatic field and ice-temperature storage technologies. The unit comprised a product storage chamber and equipment control room and was equipped with a cold storage temperature control module, electrostatic module, and heat dissipation and power supply systems. The effective sample-loading area was approximately 15 m^2^. The electric field generator operated at an input voltage of 220 V (AC) and a frequency of 50 Hz and produced an output voltage of 2500 V. All samples were positioned 30 cm from the electrostatic plate. Due to the hardware limitation of the low-voltage electrostatic field equipment, it was impossible to obtain storage conditions for the control group that were identical to those of the LVEF-treated group except for the deactivated electrostatic field. Therefore, both groups were maintained under refrigerated storage at 4 °C. The LVEF storage chamber and conventional refrigerator provided comparable temperature-controlled environments. To minimize environmental variation, identical sample preparation, packaging, storage duration, and sampling procedures were applied to both groups. These measures allowed the treatments to be evaluated under comparable refrigeration conditions. Since other regulating factors such as modified atmosphere were not involved in this study, the resulting environmental difference was relatively small. The chestnuts were stored for 15 d under both conditions.

After 15 d of storage, the chestnuts were removed, shelled, chopped, and soaked in distilled water at a sample-to-water ratio of 1:2 (*w*/*v*). The samples were homogenized using a pulping machine (PRO-12S, German Pool (Hong Kong) Co., Ltd., Hong Kong, China), and the resulting slurry was filtered through gauze to remove the solid residues. The filtrate was centrifuged at 4000× *g* for 5 min using a benchtop centrifuge (Allegra X-30R; Beckman Coulter, Brea, CA, USA). The supernatant and brown upper layer of the starch precipitate were discarded. The remaining precipitate was washed 3–4 times with distilled water and centrifuged after each wash until the supernatant was clear and free of visible impurities. The recovered starch was dried in an oven at 50 °C for 24 h. In this study, three separate portions of chestnuts were sampled from the same storage batch. Each portion underwent the above-described starch-extraction procedure independently, yielding three starch samples as three experimental replicates. The resulting samples were designated as RT-Chushuhong, RT-Changmangzi, RT-Youzhen, RT-Hongzhenzi, RT-Huangzhen, LVEF-Chushuhong, LVEF-Changmangzi, LVEF-Youzhen, LVEF-Hongzhenzi, and LVEF-Huangzhen. The prefixes RT and LVEF denote samples subjected to refrigeration treatment and low-voltage electrostatic field treatment, respectively.

### 4.3. Determination of Amylose Content

The amylose content was determined according to Guo et al. [68]. A 100 ± 0.1 mg portion of each starch sample was weighed using an analytical balance (BSA 124S, Sartorius, Germany) and dispersed in 1 mL ethanol. Subsequently, 10 mL of 1 mol/L NaOH was added, and the mixture was stirred at room temperature for 1 h until the starch completely dissolved and no visible aggregates remained. The solution was then diluted to 100 mL with distilled water. A 2 mL aliquot of the diluted solution was mixed with 50 mL of distilled water. Phenolphthalein was added as an indicator, and the solution was titrated to neutrality with 0.1 mol/L HCl until its color changed from red to colorless. Subsequently, 2 mL of 0.2% iodine solution, prepared by dissolving 2 g of KI and 0.2 g of I_2_ in 100 mL distilled water, was added. The final volume was adjusted to 100 mL with distilled water, and the solution was allowed to stand at room temperature for 30 min to ensure complete color development. Following the reported dual-wavelength protocol, the absorbance at 620 and 510 nm was measured using a UV-visible spectrophotometer (UV-1800 ultraviolet spectrophotometer, Meipuda Instruments Ltd., Shanghai, China). The amylose content was calculated using the following equation:

(1) $$\mathrm{Amylose}\left(\%\right)=\frac{\Delta A\;\mathrm{ABS}-0.01526}{0.0028}$$

where *ΔA ABS* represents the difference between the absorbance values measured at 620 and 510 nm (*ABS*_620_ − *ABS*_510_).

### 4.4. Particle Size Measurement

The particle-size distribution of chestnut starch was determined using a laser diffraction particle size analyzer (Mastersizer 2000, Malvern Panalytical, Malvern, UK) according to the procedure described by Do et al. [69]. An appropriate amount of starch was dispersed in 500 mL absolute ethanol and sonicated for 2 min. Sample obscuration was maintained at 5–10%, and the refractive index of starch was set to 1.53.

### 4.5. Determination of Water Distribution by LF-NMR

The transverse relaxation times and relative proportions of different water populations in chestnut starch powder and 6% (*w*/*w*) starch gels were measured using a low-field nuclear magnetic resonance imaging analyzer (NMZ20-060H-I; Suzhou Niomai Analytical Instrument Co., Ltd., Suzhou, China). The transverse relaxation time (*T*_2_) was measured using the Q-CPMG sequence. The *T*_2_ relaxation time distributions were obtained by multi-component inversion of the accumulated echo signals.

### 4.6. Determination of Long-Range Molecular Order by XRD

XRD patterns were recorded using an X-ray diffractometer (D8 Advance, Bruker, Karlsruhe, Germany) operated at 40 kV and 40 mA with Cu Kα_1_ radiation (λ = 0.15406 nm). The step interval was 0.02°. The areas of the crystalline and amorphous regions were quantified using PeakFit (version 4.0). The crystalline peaks were fitted using pseudo-Voigt functions, whereas the amorphous contribution was fitted using a Gaussian function [70]. The XRD-derived structural parameters were calculated using the following equations:

(2) $$\mathrm{Microcrystallineregion}\left(\%\right)=100\times \frac{I_{c1}}{I_{c1}+I_{c2}+I_{c3}}$$

(3) $$\mathrm{Subcrystallineregion}\left(\%\right)=100\times \frac{I_{c2}}{I_{c1}+I_{c2}+I_{c3}}$$

(4) $$\mathrm{Amorphousregion}\left(\%\right)=100\times \frac{I_{c3}}{I_{c1}+I_{c2}+I_{c3}}$$

where *I_c_*_1_, *I_c_*_2_, and *I_c_*_3_ represent the fitted areas of the microcrystalline, subcrystalline, and amorphous regions.

### 4.7. Determination of Short-Range Molecular Order by FTIR

The double helical structure and short-range molecular order of chestnut starch were analyzed using FTIR spectroscopy, according to the method described by Ma et al. [71]. Spectra were acquired using a VERTEX 70v spectrometer (Bruker, Ettlingen, Germany), and the samples were prepared using the KBr pressed-pellet method. Before analysis, KBr was dried at 105 °C for 8 h to eliminate the interference from residual moisture. Starch (2 mg) and KBr (200 mg) were thoroughly mixed by grinding at a 1:100 (*w*/*w*) ratio. The mixture was exposed to an infrared lamp and compressed into a thin pellet for analysis. Spectra were recorded over a range of 4000–400 cm^−1^, at a resolution of 4 cm^−1^, using 32 accumulated scans. Gaussian deconvolution analysis of all spectra was performed using OMNIC software (version 8.0, Nicolet, WI, USA) to calculate the intensity ratios of the peaks at 995/1022 cm^−1^ and 1047/1022 cm^−1^.

### 4.8. Determination of Pasting Properties by RVA

The pasting properties of chestnut starch were determined using a rapid viscosity analyzer (RVA 4500; Perten Co., Ltd., Hägersten, Sweden). A 6% (*w*/*w*) starch suspension was prepared using distilled water. The RVA temperature and mixing program was as follows: 0–10 s at 50 °C and 960 rpm; 10–60 s at 50 °C and 160 rpm; 60–285 s, heat to 95 °C at 12 °C/min and 160 rpm; 285–435 s at 95 °C and 160 rpm; 435–660 s, cool to 50 °C at −12 °C/min and 160 rpm; 660–780 s at 50 °C and 160 rpm. Five parameters were recorded from the viscoelasticity graph: peak viscosity, trough viscosity, final viscosity, peak time, and gelatinization temperature. Two additional parameters were calculated: the breakdown and setback viscosities. The viscosity profile was plotted as a function of temperature and time, and the resulting characteristic values were used to evaluate the pasting properties of each sample [72].

### 4.9. Differential Scanning Calorimetry (DSC)

The thermal properties of chestnut starch were analyzed using differential scanning calorimetry, according to the method described by Zhong et al. [73]. Starch (30 mg) was weighed directly into a DSC pan, and 100 μL of distilled water was added. The pan was sealed and equilibrated for 12 h prior to the analysis. Measurements were performed using a differential scanning calorimeter (DSC 250, TA Instruments, New Castle, DE, USA). The samples were heated from 10 to 160 °C at a rate of 5 °C/min. The gelatinization and retrogradation behaviors of each sample were evaluated using DSC thermograms. The onset temperature (*T*_0_), peak temperature (*T_p_*), conclusion temperature (*T_c_*), and gelatinization enthalpy (*ΔH)* were obtained from the first heating thermograms.

### 4.10. Texture Profile Analysis (TPA)

Following RVA, the resulting chestnut starch gels were subjected to texture profile analysis using a TA-XT2i texture analyzer (TA-XT2i texture analyzer, Stable Micro Systems, Godalming, UK). A P/36R probe was used with a pre-test speed of 1 mm/s, test speed of 2 mm/s, post-test speed of 1 mm/s, trigger force of 5 g, and a 5 s interval between the two compression cycles. The parameters evaluated were hardness, springiness, cohesiveness, chewiness, and resilience.

### 4.11. Determination of In Vitro Starch Digestibility

In vitro starch digestibility was determined using a previously described method with modifications [74]. A mixed-enzyme digestion solution containing 1200 U of α-amylase and 960 U of glucoamylase was prepared in 0.01 M phosphate-buffered saline (PBS; pH 6.5). To determine the initial glucose content, 20 mg of starch was mixed with 1 mL of 0.1 mol/L sodium acetate-acetic acid buffer (pH 5.2) and equilibrated in a water bath at 37 °C for 10 min. The suspension was subsequently heated in a boiling-water bath for 5 min and centrifuged at 9500× *g* for 10 min. The glucose content of the resulting supernatant was considered as the 0 min hydrolysate (G_0_). For enzymatic digestion, 20 mg aliquots of starch were mixed with 1 mL of the mixed enzyme solution and 1 mL of sodium acetate-acetic acid buffer. The reaction mixtures were incubated in a shaking water bath at 37 °C and 150 rpm. Separate mixtures were collected after 20 and 120 min. The enzymatic reaction was terminated by heating the samples in a boiling water bath for 15 min, followed by centrifugation at 9500× *g* for 10 min. The glucose contents of the collected supernatants were designated as G_20_ and G_120_, respectively. The glucose content was determined using the DNS colorimetric method. The absorbance was measured at 530 nm using a spectrophotometer. The RDS, SDS, and RS contents were calculated using the following equations:

(5) $$\mathrm{RDS}(\%)\;=\;\frac{(G_{20}-G_{0})\;\times \;0.9}{\mathrm{TS}}\;\times \;100\%$$

(6) $$\mathrm{SDS}(\%)=\frac{(G_{120}-G_{20})\;\times \;0.9}{\mathrm{TS}}\;\times \;100\%$$

RS(%) = TS − RDS − SDS(7)

where *G*_0_, *G*_20_, and *G*_120_ represent the glucose contents measured at 0, 20, and 120 min, respectively, and TS represents the total starch content.

### 4.12. Statistical Analysis

Statistical analyses were performed using the IBM SPSS Statistics 27.0.1. Data are presented as mean ± standard deviation and visualized using Origin software (2021 version, OriginLab Corporation, Northampton, MA, USA). Differences were evaluated using one-way analysis of variance (ANOVA), followed by Duncan’s post hoc test. All measurements were performed in triplicate.

## 5. Conclusions

This study provides the first comparative assessment of the effects of refrigeration treatment (RT) and low-voltage electrostatic field (LVEF) treatment on the starch structure, pasting properties, and in vitro digestibility of five representative *C. henryi* cultivars from different regions of China. These findings suggest that LVEF may weaken hydrogen bonds within starch molecules, allowing unfolded starch chains to rearrange into more ordered molecular configurations and denser hydrogen bond networks under low-temperature conditions, thereby increasing crystallinity and molecular order. This molecular reorganization may also restrict starch swelling, as reflected by the lower PV value. In addition, LVEF generally reduced the free water content, breakdown viscosity (BV), and setback viscosity (SV) of chestnut starch, while increasing its water-holding capacity and resistance to shear and retrogradation, and improving the textural properties of starch gels. In contrast to the other cultivars, LVEF-treated Chushuhong starch had a higher amylose content, smaller particle size, and higher PV than its RT counterpart. LVEF-treated Chushuhong and Changmangzi starches exhibited favorable processing properties but faster digestion rates under in vitro conditions. Collectively, these findings indicate that the effects of LVEF on *C. henryi* starch are dependent on the cultivar. This study provides a basis for the appropriate application of LVEF and the development of *C. henryi* starch-based products.

## Figures and Tables

**Table 1:** Pasting property parameters of chestnut starch isolated from five cultivars.

Index	Storage Method	Chushuhong	Changmangzi	Youzhen	Hongzhenzi	Huangzhen
Pasting temperature (°C)	RT	93.9 ± 0.08 ^b^	90.7 ± 0.19 ^c^	94.6 ± 0.13 ^a^	94.8 ± 0.21 ^a^	94.7 ± 0.2 ^a^
	LVEF	93.3 ± 0.2 ^c,^*	93.2 ± 0.04 ^c,^*	95.2 ± 0.06 ^a,^*	79.3 ± 0.13 ^d,^*	94.7 ± 0.07 ^b^
Peak viscosity (cP)	RT	581 ± 2.19 ^e^	727 ± 3.14 ^a^	614 ± 2.62 ^c^	604 ± 3.57 ^d^	674 ± 2.03 ^b^
	LVEF	801 ± 1.12 ^a,^*	577 ± 0.28 ^d,^*	593 ± 1.57 ^c,^*	602 ± 3.41 ^b^	592 ± 3.38 ^c,^*
Breakdown viscosity (cP)	RT	154 ± 0.22 ^b^	187 ± 0.1 ^a^	147 ± 0.58 ^c^	129 ± 0.3 ^e^	131 ± 0.7 ^d^
	LVEF	119 ± 0.32 ^e,^*	160 ± 0.43 ^b,^*	137 ± 0.43 ^c,^*	166 ± 1.14 ^a,^*	127 ± 0.04 ^d,^*
Setback viscosity (cP)	RT	375 ± 1.03 ^b^	442 ± 0.45 ^a^	295 ± 0.71 ^c^	177 ± 0.63 ^e^	264 ± 1 ^d^
	LVEF	186 ± 0.61 ^e,^*	301 ± 1.12 ^a,^*	299 ± 1.05 ^b,^*	221 ± 1 ^d,^*	261 ± 1.1 ^c,^*

Mean of three measurements ± standard deviation (SD). Values for the same storage method and different chestnut starches with different letters are significantly different (*p* < 0.05). * indicates a statistically significant difference between the two groups within the same variety (*p* < 0.05). (RT: refrigerated treatment; LVEF: low-voltage electrostatic field).

**Table 2:** Particle size distribution (PSD), water distribution, XRD pattern and selected FTIR peak ratios characteristics of chestnut starch isolated from five cultivars.

Index		Storage Method	Chushuhong	Changmangzi	Youzhen	Hongzhenzi	Huangzhen
PSD	D [2,3] (μm)	RT	10.53 ± 0.49 ^a^	9.54 ± 0.06 ^b^	7.50 ± 0.08 ^c^	9.23 ± 0.29 ^b^	9.05 ± 0.19 ^b^
		LVEF	8.47 ± 0.08 ^c,^*	10.53 ± 0.25 ^a,^*	8.5 ± 0.20 ^c,^*	9.8 ± 0.24 ^b,^*	8.63 ± 0.14 ^c,^*
LF-NMR	A21 (%)	RT	90.40 ± 0.39 ^a^	90.24 ± 0.25 ^a^	90.38 ± 0.52 ^a^	90.21 ± 0.22 ^a^	90.01 ± 0.22 ^a^
		LVEF	89.91 ± 0.20 ^a^	90.47 ± 0.51 ^a^	89.12 ± 0.47 ^b,^*	90.45 ± 0.38 ^a^	90.16 ± 0.22 ^a^
	A22 (%)	RT	8.84 ± 0.34 ^a^	9.05 ± 0.26 ^a^	8.96 ± 0.49 ^a^	9.12 ± 0.17 ^a^	9.32 ± 0.18 ^a^
		LVEF	9.48 ± 0.19 ^b,^*	8.88 ± 0.47 ^b^	10.24 ± 0.44 ^a,^*	8.84 ± 0.35 ^b^	9.17 ± 0.21 ^b^
	A23 (%)	RT	0.76 ± 0.06 ^a^	0.72 ± 0.02 ^a^	0.66 ± 0.05 ^a^	0.67 ± 0.06 ^a^	0.67 ± 0.05 ^a^
		LVEF	0.62 ± 0.02 ^b^	0.65 ± 0.04 ^ab,^*	0.64 ± 0.03 ^b^	0.71 ± 0.05 ^a^	0.67 ± 0.002 ^ab^
XRD	Proportion of the microcrystalline region (%)	RT	26.74 ± 0.11 ^b^	33.79 ± 0.02 ^a^	24.76 ± 0.17 ^d^	26.33 ± 0.16 ^c^	26.31 ± 0.10 ^c^
		LVEF	35.12 ± 0.06 ^a,^*	33.44 ± 0.05 ^b,^*	25.77 ± 0.14 ^d,^*	29.21 ± 0.14 ^c,^*	29.37 ± 0.12 ^c,^*
	Proportion of the sub-crystalline region (%)	RT	51.20 ± 0.11 ^b^	49.52 ± 0.35 ^c^	49.05 ± 0.21 ^c^	53.72 ± 0.29 ^a^	49.45 ± 0.03 ^c^
		LVEF	51.61 ± 0.41 ^b^	46.80 ± 0.24 ^e,^*	52.72 ± 0.23 ^a,^*	50.91 ± 0.27 ^c,^*	48.28 ± 0.29 ^d^
	Proportion of the non-crystalline region (%)	RT	22.06 ± 0.20 ^c^	16.69 ± 0.09 ^e^	26.19 ± 0.09 ^a^	19.95 ± 0.15 ^d^	24.24 ± 0.11 ^b^
		LVEF	13.27 ± 0.09 ^d,^*	19.76 ± 0.08 ^c,^*	21.51 ± 0.11 ^b,^*	19.88 ± 0.09 ^c^	22.35 ± 0.20 ^a,^*
FTIR	R1047/1022	RT	0.785 ± 0.008 ^c^	0.870 ± 0.004 ^a^	0.745 ± 0.019 ^d^	0.830 ± 0.008 ^b^	0.845 ± 0.009 ^b^
		LVEF	0.802 ± 0.005 ^c,^*	0.850 ± 0.006 ^a,^*	0.813 ± 0.008 ^b,^*	0.852 ± 0.005 ^a,^*	0.859 ± 0.003 ^a^
	R995/1022	RT	0.846 ± 0.002 ^d^	1.056 ± 0.002 ^a^	0.897 ± 0.001 ^c^	1.034 ± 0.015 ^b^	1.054 ± 0.011 ^a^
		LVEF	0.967 ± 0.013 ^d,^*	1.096 ± 0.019 ^b,^*	0.968 ± 0.011 ^d,^*	1.046 ± 0.017 ^c^	1.159 ± 0.023 ^a,^*

Mean of three measurements ± standard deviation. Values for the same storage method and different chestnut starch with different letters are significantly different (*p* < 0.05). * indicates a statistically significant difference between the two groups among the same varieties (*p* < 0.05). (RT: refrigerated treatment; LVEF: low-voltage electrostatic field).

**Table 3:** Thermal properties of chestnut starch isolated from five cultivars.

Index		Storage Method	Chushuhong	Changmangzi	Youzhen	Hongzhenzi	Huangzhen
The first peak	*T_p_* (°C)	RT	65.7 ± 0.23 ^a^	62.5 ± 0.43 ^b^	31.1 ± 0.18 ^e^	48.8 ± 0.25 ^d^	56.5 ± 0.4 ^c^
		LVEF	39.6 ± 0.15 ^d,^*	60.1 ± 0.33 ^b,^*	—	49.8 ± 0.16 ^c,^*	49.7 ± 0.26 ^c,^*
	*ΔH* (J/g)	RT	18.3 ± 0.03 ^a^	6.1 ± 0.03 ^d^	2.5 ± 0.01 ^e^	12.8 ± 0.10 ^c^	15.0 ± 0.01 ^b^
		LVEF	7.9 ± 0.03 ^d,^*	12.7 ± 0.07 ^b,^*	—	15.4 ± 0.08 ^a,^*	9.6 ± 0.03 ^c,^*
The second peak	*T_p_* (°C)	RT	116.5 ± 0.66 ^b^	130.4 ± 0.73 ^a^	104.4 ± 0.73 ^d^	105.2 ± 0.21 ^d^	114.7 ± 0.21 ^c^
		LVEF	112.4 ± 0.16 ^b,^*	117.3 ± 0.64 ^a,^*	117.1 ± 0.12 ^a,^*	109.6 ± 0.21 ^c,^*	107.7 ± 0.34 ^d,^*
	*ΔH* (J/g)	RT	7.2 ± 0.05 ^b^	20.6 ± 0.12 ^a^	5.1 ± 0.03 ^d^	5.4 ± 0.03 ^c^	5.1 ± 0.02 ^d^
		LVEF	5.9 ± 0.03 ^c,^*	6.2 ± 0.02 ^c,^*	29.5 ± 0.11 ^a,^*	6.3 ± 0.02 ^b,^*	5.6 ± 0.03 ^d,^*

The first “—“ indicates no distinct peak for this sample. The second “—“ denotes that reliable quantification cannot be achieved. Mean of three measurements ± standard deviation. Values for the same storage method and different chestnut starch with different letters are significantly different (*p* < 0.05). * indicates a statistically significant difference between the two groups within the same variety (*p* < 0.05). (RT: refrigerated treatment; LVEF: low-voltage electrostatic field).

**Table 4:** Water distribution and texture properties of chestnut starch gel from five cultivars.

Index		Storage Method	Chushuhong	Changmangzi	Youzhen	Hongzhenzi	Huangzhen
LF-NMR	*A*_21_ (%)	RT	12.26 ± 0.38 ^c^	13.63 ± 1.23 ^bc^	15.60 ± 1.51 ^ab^	14.81 ± 0.96 ^ab^	16.89 ± 1.38 ^a^
		LVEF	13.94 ± 0.58 ^b,^*	13.67 ± 1.11 ^b^	15.20 ± 1.11 ^ab^	15.44 ± 0.88 ^ab^	16.46 ± 1.60 ^a^
	*A*_22_ (%)	RT	1.95 ± 0.10 ^b^	2.39 ± 0.44 ^b^	3.64 ± 0.51 ^a^	3.32 ± 0.28 ^a^	3.51 ± 0.84 ^a^
		LVEF	2.47 ± 0.09 ^b,^*	2.33 ± 0.45 ^b^	3.20 ± 0.09 ^a^	3.24 ± 0.29 ^a^	2.75 ± 0.34 ^ab^
	*A*_23_ (%)	RT	85.79 ± 0.40 ^a^	83.98 ± 1.27 ^ab^	80.76 ± 1.83 ^c^	81.87 ± 1.15 ^bc^	79.60 ± 2.04 ^c^
		LVEF	83.59 ± 0.66 ^ab,^*	84.00 ± 1.08 ^a^	81.60 ± 1.14 ^bc^	81.32 ± 0.74 ^c^	80.80 ± 1.79 ^c^
TPA	Hardness (N)	RT	58.29 ± 2.74 ^e^	133.57 ± 9.32 ^a^	76.60 ± 5.44 ^b^	65.77 ± 2.93 b^c^	63.93 ± 0.72 ^d^
		LVEF	194.33 ± 15.34 ^a,^*	160.50 ± 2.39 ^b,^*	64.07 ± 1.08 ^d,^*	57.70 ± 4.25 ^d,^*	141.21 ± 8.61 ^c,^*
	Springness	RT	0.95 ± 0.03 ^ab^	1.01 ± 0.06 ^a^	0.93 ± 0.01 ^b^	0.91 ± 0.01 ^b^	0.78 ± 0.01 ^c^
		LVEF	0.95 ± 0.02 ^a^	0.97 ± 0.00 ^a^	0.94 ± 0.03 ^a^	0.85 ± 0.02 ^b,^*	0.93 ± 0.01 ^a,^*
	Cohesiveness	RT	0.74 ± 0.04 ^ab^	0.91 ± 0.03 ^a^	0.75 ± 0.05 ^ab^	0.75 ± 0.03 ^ab^	0.62 ± 0.01 ^b^
		LVEF	0.89 ± 0.02 ^a,^*	0.92 ± 0.03 ^a^	0.70 ± 0.04 ^c^	0.68 ± 0.04 ^c,^*	0.82 ± 0.01 ^b,^*
	Chewiness (N)	RT	41.02 ± 2.7 ^b^	123.08 ± 16.50 ^a^	54.04 ± 10.81 ^b^	45.02 ± 3.59 ^b^	32.61 ± 1.03 ^c^
		LVEF	164.30 ± 16.07 ^a,^*	143.50 ± 5.38 ^b,^*	42.23 ± 1.37 ^d,^*	33.30 ± 2.73 ^d,^*	108.57 ± 8.45 ^c,^*
	Resilience	RT	0.45 ± 0.02 ^b^	0.68 ± 0.03 ^a^	0.51 ± 0.04 ^b^	0.49 ± 0.02 ^b^	0.37 ± 0.01 ^c^
		LVEF	0.67 ± 0.04 ^b,^*	0.74 ± 0.04 ^a,^*	0.47 ± 0.01 ^d,^*	0.33 ± 0.03 ^e,^*	0.59 ± 0.02 ^c,^*

Mean of three measurements ± standard deviation. Values for the same storage method and different chestnut starch gels with different letters are significantly different (*p* < 0.05). * indicates a statistically significant difference between the two groups among the same varieties (*p* < 0.05). (RT: refrigerated treatment; LVEF: low-voltage electrostatic field).

**Table 5:** The content of RS, SDS and RDS of chestnut starch isolated from five cultivars.

Index	Storage Method	Chushuhong	Changmangzi	Youzhen	Hongzhenzi	Huangzhen
RS (%)	RT	13.15 ± 0.05 ^e^	22.77 ± 0.06 ^c^	13.95 ± 0.2 ^d^	45.95 ± 0.71 ^a^	30.62 ± 0.26 ^b^
	LVEF	9.25 ± 0.09 ^e,^*	19.77 ± 0.27 ^c,^*	18.24 ± 0.25 ^d,^*	27.54 ± 0.26 ^a,^*	23.48 ± 0.19 ^b,^*
SDS (%)	RT	12.7 ± 0.13 ^c^	1.46 ± 0.01 ^d^	24.12 ± 0.2 ^b^	0.76 ± 0.01 ^e^	29.99 ± 0.19 ^a^
	LVEF	21.26 ± 0.22 ^a,^*	15.4 ± 0.05 ^d,^*	17.77 ± 0.07 ^c,^*	18.55 ± 0.06 ^b,^*	8.52 ± 0.09 ^e,^*
RDS (%)	RT	74.74 ± 0.45 ^b^	76.58 ± 0.77 ^a^	61.67 ± 0.52 ^c^	52.97 ± 0.33 ^d^	39.19 ± 0.13 ^e^
	LVEF	70.04 ± 0.5 ^a,^*	64.88 ± 0.88 ^c,^*	64.11 ± 1.2 ^c,^*	53.88 ± 0.48 ^d,^*	68.45 ± 0.39 ^b,^*

Mean of three measurements ± standard deviation. Values for the same storage method and different chestnut starch with different letters are significantly different (*p* < 0.05). * indicates a statistically significant difference between the two groups among the same varieties (*p* < 0.05). (RT: refrigerated treatment; LVEF: low-voltage electrostatic field).

## Data Availability

Data will be made available on request.

## Supplementary Materials

The following supporting information can be downloaded at: https://www.mdpi.com/article/10.3390/molecules31213900/s1, Table S1: Basic information about the chestnut.
