# Supplementary material for: The Relationship of Cultivars, Structures and In Vitro Digestibility of Chinese Chestnut (Castanea henryi) Starches Treated with Low-Voltage Electrostatic Field Technology
Source: Molecules. 2026 Oct 29;31(21):3900. doi: 10.3390/molecules31213900 (PMC13765514; doi:10.3390/molecules31213900)
Supplement: Supplementary file 1 [file molecules-31-03900-s001.zip › molecules-4609443-supplementary.pdf]

Table S1 Basic information about the chestnut.

Variety Name	Place of origin	eggshell	Fruit	Flesh	Flavor	Maturity stage
Chushuhong	Lishui,Zhejiang, China	Reddish-brown, glossy.	Medium-sized, standard conical shape, the top of the fruit is relatively full.	Good glutinous texture.	Sweet and fragrant taste.	From late August to early September is one of the earliest varieties of conical chestnuts on the market.
Changmangzi	Jianou,Fujian, China	Very large, dark-colored, with long and dense spines.	Relatively large, the weight of a single nut is considered large among conical chestnuts.	The glutinous texture is not as good as that of Huangzhen and Youzhen.	The sweetness is not as high as that of Huangzhen and Youzhen, but the accumulation of flavor substances is more sufficient.	After mid-October, or even later.
Youzhen	Jianou,Fujian, China	The color is dark brown, glossy, and lustrous.	The fruit has a rounded conical base, a standard shape, and the size of the grains is usually medium to large, plump and uniform.	It has a very strong glutinous texture, with a delicate and soft mouthfeel, almost without any graininess.	The aroma is very rich, the sweetness is high, and the flavor is pure.	From the end of September to mid-October.
Hongzhenzi	Jianou,Fujian, China	Reddish-brown	smaller	Tenderer	Higher sweetness	Mid-September
Huangzhen	Jianou,Fujian, China	Reddish-brown in color, glossy, with minimal fuzz.	Round at the base and conical at the top, standard and beautiful in shape, uniform in size.	Glutinous, neither dry nor hard.	High in sweetness, with a pure aroma.	From mid to late September to early October
